# Etiology and Management of Behavioral Disorder in Adults With Intellectual and Developmental Disabilities

**DOI:** 10.7759/cureus.14221

**Published:** 2021-03-31

**Authors:** Govind H Kallumkal, Rafik Jacob, Linda Edwards

**Affiliations:** 1 Internal Medicine, University of Florida College of Medicine, Gainesville, USA; 2 Internal Medicine, University of Florida College of Medicine – Jacksonville, Jacksonville, USA

**Keywords:** intellectual and developmental disabilities, antipsychotics, behavioral modification, behavioral disorder

## Abstract

Intellectual disability (ID) encompasses a wide variety of disorders that can severely affect an individual’s cognitive, social, emotional, and physical development, even when identified early in life. Initially, individuals with such disorders had shorter life expectancies. However, medical advances have increased the life expectancy of individuals with ID similar to that of the general population. More attention must be paid to manage diseases affecting the intellectually disabled elderly, such as diabetes, cardiovascular disease, chronic constipation, and behavioral disorders.

## Introduction and background

An issue of particular significance for caretakers and physicians of individuals with intellectual disability (ID) is behavioral disorder/disruption. Ranging from mild repetitive movements to actions injurious to caretakers and self, behavioral disorders can be challenging for caretakers. Often, the etiology of these disruptions is secondary to alterations in the patient’s environment and can be caused by physical and psychiatric health issues. The patient’s communication difficulties can make the identification of the etiology of the behavioral disorder challenging. Treatment is multidisciplinary and requires the use of therapy and antipsychotics.

## Review

Etiology of behavioral disorder in ID

Behavioral disruption ranges from minor stereotypical actions, such as body rocking, hand wringing, and crossing of legs, to behavior injurious to self, others, and property [1]. The Royal College of Psychiatrists has defined behavioral disruption as “it is of such intensity, frequency, or duration, as to threaten the quality of life and/or the physical safety of the individual or others, and is likely to lead to responses that are restrictive, aversive, or result in exclusion.” The prevalence of behavioral disorders ranges between 10% and 55% [2,3]. It is estimated that dangerous, self-injurious actions occur at a rate of 4-5% in children [4]. Additionally, the severity of ID plays a significant role in the severity of behavioral disruption. The worse the disability, the worse the disruption [5].

The surroundings and environment often play a role in behavioral disruption. In circumstances where the patients feel uncomfortable, he/she is more likely to lash out in frustration/discomfort [4]. For instance, if a physician’s office represents a disturbing environment for the patient, they may respond with disruptive behavior. These behaviors may also be secondary to frustration with the caregiver demands [6]. Certain patients may respond poorly if they are not agreeable to caretaker requests to perform a particular exercise or eat a certain food. Depending on the severity of the reaction, caregivers may attempt to placate the patients to stop the behavior. They often do so by continuing to allow performance of actions that are not desired or by supplying treats or toys. However, placation can create a positive feedback response, which further reinforces poor behavior [7]. As patients begin to associate desirable consequences to their problem behavior, they are further empowered in their actions.

Anxiety and other comorbid psychiatric disorders are a significant concern when caring for individuals with ID [8]. A total of 55.3% of children with non-specified developmental disorders and 26.8% of adults with ID show symptoms of anxiety. Similarly, 4.4% of individuals with ID exhibit depression [9]. Comorbid disorders, which are difficult to diagnose in these populations, can increase feelings of discomfort, further contributing to behavioral disruption. Psychiatric issues are difficult to diagnose in individuals who cannot communicate adequately. This can often lead to feelings of increased frustration by the patients and can be an added reason for disruptive behavior.

Physical discomfort can also cause behavioral disruption, as seen in emotional and psychiatric discomfort [10]. The reduced capability of self-expression often prolongs discomfort and raises the frustration associated with it. One prevalent example is constipation. Up to 75% of individuals with ID experience chronic constipation [10-11], which is associated with increased behavioral disruption [12]. Other chronic issues, such as gastroesophageal reflux disease (GERD), occur in 48% of individuals with ID [13]. Common chronic conditions, including allergies [14] and otitis media [15], can also cause problem behavior [16]. Any medical issue that causes significant discomfort to patients can lead to problematic behavior. Dysmenorrhea is twice as common in individuals with ID [17]. If behavioral disruption is associated with dysmenorrhea, the problematic behavior is also cyclic, resolving with the end of menstruation [18]. Other issues, such as undiagnosed physical trauma, seizure disorder, and vision and hearing impairment, can be other stimuli for problematic behavior [10].

Behavioral disorders may also be secondary to physical and sexual abuse. Individuals with ID are abused at significantly higher rates than the general population. Up to 34% of individuals with ID experience sexual abuse [19]. Furthermore, these individuals are more likely to experience issues, such as depression or anxiety, and are more likely to participate in self-injury or stereotypy [20-22].

Diagnosis of behavioral disorders in individuals with ID

The diagnosis of behavioral disruption and its etiology begins with a thorough history, including baseline behavior, identification of events that initiate problem behavior, and experiences that reinforce or inhibit problem behavior [23]. Particular focus should also be given to understanding the caretaker’s response to disruptive behavior. Because patients may have difficulty in expression and communication, histories must often be taken from caregivers [24]. A significant understanding of behavioral disruption may be obtained through rating scales, interviews, and observations in natural settings.

In case of behavioral disorder secondary to physical discomfort, a thorough history of events before and after the behavioral changes, and an adequate physical examination and imaging, may be warranted [10]. For example, if a behavioral change is secondary to abdominal discomfort from a history of constipation, a thorough abdominal and rectal examination along with radiography may be warranted. Furthermore, patients' actions can help identify the nature of their pain. Ear poking or pulling may occur concurrently with otitis media [25], hand mouthing with GERD [26], and headbanging with allergies [27].

Management of behavioral disorders in individuals with ID

Therapy and behavioral modifications are the foundations of behavioral disruption management. Therapy should be attempted before the use of antipsychotics when managing behavioral disruptions. The primary modality of therapy is function-based treatment [28]. The goal of the specific therapy is to reinforce or support appropriate alternative behaviors while negatively reinforcing bad behavior and reducing its frequency. Early therapy initiation is more likely to eliminate bad behavior [20]. Appropriate behavior is more likely to be preserved when caretakers maintain adequate reinforcement [7]. Resolving medical and metabolic contributions is warranted in conjunction with behavioral therapy. Using both modalities of care will allow the existing stimulus to be resolved, while equipping patients and caretakers with better coping techniques.

Antipsychotics may be necessary when therapy cannot eliminate behavioral disruption adequately or when behavior poses a risk to patients or their caregivers [29]. Medications should be used to decrease emotional creativity, irritability, impulsivity, and work synergistically with therapy. Among all patients with ID, approximately 30% regularly use antipsychotics [30] compared to 48% of patients with ID who exhibit significant behavioral problems [31]. The two most frequently used medications are risperidone and aripiprazole, both are administered in a dose lower than that in schizophrenia treatment [32]. These medications are commonly used for the management of ID. Efficacy is unclear when used in managing problem behavior. Previous studies show mixed efficacy when compared to placebo [33-36]. However, treatment is significantly more effective when used for patients with comorbid psychiatric disorders [32].

Antipsychotics medications should be administered with caution because of extensive side effects, particularly in individuals with ID [37]. In case of patients on risperidone, physicians must pay close attention to extrapyramidal symptoms (occurring in 37.6% of users) such as tremor, dystonia, and akathisia. Metabolic changes, such as hyperprolactinemia (occurring in 87.2% of users), weight gain, hyperglycemia, hyperlipidemia, and other effects such as constipation and amenorrhea, should be closely monitored [38]. Aripiprazole's side effect profile has some similarities to risperidone, including, but not limited to, restlessness/akathisia, somnolence, and nausea in a dose-dependent manner [39]. The long-term metabolic side effects of these medications should be considered owing to the sedentary nature of many individuals with IDs. Hyperprolactinemia can lead to hypogonadism and osteoporosis, while hyperlipidemia and hyperglycemia can lead to an increased cardiovascular risk [40].

When atypical antipsychotics are initiated, baseline vital signs, such as blood pressure and waist circumference, should be obtained in addition to laboratory findings, such as fasting lipids, glucose, thyroxine, and thyroid-stimulating hormone levels. Plasma drug levels need not be monitored [41]. However, patients should be monitored monthly to assess body weight, with laboratory findings being evaluated every three months. If laboratory findings and vital signs remain stable, follow-up may be less frequent [42]. Once behavioral issues are appropriately controlled, medications should be weaned to their smallest effective dose [43].

## Conclusions

With the improvement in medical care, the life expectancy of patients with ID has increased, and their care has become similar to that of the general aging population. Problematic or disruptive behavior is one of the most common and challenging issues faced by physicians and caretakers. Actions range from benign, such as ear tugging, to behaviors that are dangerous to patients and caretakers. Inappropriate coping mechanisms cause actions in response to psychiatric and medical distress, and environmental change. Diagnosis requires thorough history taking, appropriate physical examinations, and imaging when required. Treatment focuses on resolving the distressing stimulus while reinforcing proper coping techniques. Antipsychotics, such as risperidone and aripiprazole, can be used in circumstances where problem behavior is refractory to therapy. Patients managed with such medications should be monitored regularly for possible side effects, particularly because of the high-risk status of individuals with ID.
